# Outcomes of continuous flow left ventricular assist device after surgical left ventricular restoration

**DOI:** 10.1007/s11748-023-01917-8

**Published:** 2023-02-21

**Authors:** Takayuki Gyoten, Minoru Ono

**Affiliations:** Department of Cardiac Surgery, The University of Tokyo, 7-3-1 Hongo, Bunkyo-Ku, Tokyo 113-8655 Japan

**Keywords:** Continuous flow left ventricular assist device implantation, Surgical left ventricular restoration, Endoventricular Dacron patch, Posterior restoration procedure, Septal anterior ventricular exclusion, Endoventricular circular patch plasty

## Abstract

**Objectives:**

This study aimed to report the clinical outcomes of continuous flow left ventricular assist device implantation in end-stage chronic heart failure patients with a history of surgical left ventricular restoration.

**Methods:**

We retrospectively identified 190 patients undergoing continuous flow left ventricular assist device implantation at our center from November 2007 to April 2020. In total, six patients underwent continuous flow left ventricular assist device implantation after various types of surgical left ventricular restoration procedures, including endoventricular circular patch plasty (*n* = 3), posterior restoration procedure (*n* = 2), and septal anterior ventricular exclusion (*n* = 1).

**Results:**

Continuous flow left ventricular assist device (Jarvik 2000, *n* = 2; EVAHEART, *n* = 1; HeartMate II, *n* = 1; DuraHeart, *n* = 1; HVAD, *n* = 1) was successfully implanted in all patients. During a median follow-up of 48 months (interquartile range, 39–60 months; censoring heart transplantation), no death was documented, which means that overall survival after left ventricular assist device implantation was 100% at any time point. Finally, three patients received heart transplantation (waiting time: 39, 56, and 61 months, respectively) and the other three patients are still awaiting heart transplantation (waiting time: 12, 41, and 76 months, respectively).

**Conclusions:**

In our series, continuous flow left ventricular assist device implantation after surgical left ventricular restoration was safe and feasible, even if an endoventricular patch was used, and effective for bridge to transplant strategy.

**Supplementary Information:**

The online version contains supplementary material available at 10.1007/s11748-023-01917-8.

## Introduction

Surgical ventricular restoration (SVR) has been developed for patients with end-stage heart failure which is refractory to guideline-directed heart failure therapy in Japan, the United States of America, and Europe, owing to a shortage of donors [1–4]. SVR procedures modify the left ventricular shape with ventricular volume reduction, entailing resection or exclusion of stiff segments of akinetic scar or relatively compliant dyskinetic scar [5].

The landmark Surgical Treatment for Ischemic Heart Failure (STICH) trial showed no benefit of SVR from the standpoint of the overall survival in ischemic heart disease. However, some researchers believe that the STICH trial may have failed to show an improvement in survival by SVR because the surgery did not make the ventricle small enough [5, 6]. A small number of hospitals have still continued to perform SVR, particularly in Italy and Japan. According to previous reported studies, the rates of survival, and recurrent heart failure were acceptable after SVR; survival rate of 82.4% at 8 years in the ischemic cardiomyopathy group and postoperative New York Heart Association (NYHA) class ≤ II, more than 80% at a mean follow-up period of 3 years and 66% at 8 years in the dilated cardiomyopathy group [5, 7].

However, some patients may develop heart failure after successful SVR at later follow-up [8]. This phenomenon is mainly caused by recurrent remodeling and reduced contraction of the left ventricle.

During the past decades, a continuous flow left ventricular assist device (Cf-LVAD) therapy has been a treatment option in advanced heart failure, with improving outcomes around the world [9, 10]. In particular, modern generation LVADs are relatively small implantable centrifugal pumps, and the successful attribution boosted the number of devices implantation as bridge to transplant (BTT) and destination therapy strategy [11–13]. At present, cf-LVAD implantation seems to be a feasible option as a life-saving procedure for recurrent heart failure with failed SVR along with heart transplantation.

However, an efficacy and feasibility of VAD therapy were barely investigated, due to scarce clinical experience, and there are no clear recommendations for patients with a prior history of SVR [14, 15]. Cf-LVAD implantation may be challenging because these patients have a complex left ventricular apical anatomy that may hinder the decision of optimal inflow cannula position and angle. This is because cf-LVAD implantation in some patients after SVR with patch repair may require removal of the patch and reconstruction of the left ventricle for the proper position and direction of a left ventricular inflow cannula.

The aim of the present study was to review our experience with cf-LVAD therapy in patients with a prior SVR and provide clinical outcome and follow-up data, including mortality and adverse events.

## Methods

### Data collection and follow-up

This single-center study was approved by Institutional Ethics Committee of the University of Tokyo (3031-[4]). We included all patients from our institutional database qualifying for LVAD implantation for recurrent severe heart failure after SVR (SVR was performed in other hospitals). Clinical decisions were made in an interdisciplinary heart team conference consisting of cardiologists, cardiac surgeons, perfusionists, cardio-anesthesiologists, and VAD coordinators. As a bridge to transplant, a total of 190 patients were implanted with durable LVADs at our center between November 2007 and April 2020. All types of devices have been included in this study, including the DuraHeart (Terumo Heart, Ann Arbor, MI, USA), EVAHEART (Sun Medical technology Research Corp, Nagano, Japan), Jarvik 2000 (Jarvik Heart, New York, NY), HVAD (Medtronic, USA), and HeartMate II (Abbott Medical, Abbott Park, USA). Demographics and clinical data also include invasive pressures recorded by right heart catheterization (RHC). We also collected data on imaging tests, laboratory values, and surgical factors. Transthoracic echocardiography (TTE) was regularly performed before LVAD implantation, 1 and 12 months after LVAD implantation, and subsequently every year. Doppler images were judged as follows: 1 +  = mild, 2 +  = mild-to-moderate, 3 +  = moderate-to-severe, and 4 +  = severe [16]. The clinical follow-up period ended on April 30, 2021, when the last enrolled patient had completed 1 year of the follow-up period. The end-points of the study were first re-hospitalization for heart failure or cardiovascular death. Re-hospitalization for heart failure was defined as new-onset or worsening signs and symptoms of heart failure that required urgent therapy resulting in hospitalization.

### Types of left ventricular restoration and concomitant procedures

The SVR procedures, including endoventricular circular patch plasty (EVCPP), septal anterior ventricular exclusion (SAVE), and posterior restoration procedure (PRP), were selected and performed based on the affected region of the ventricle and the surgeon’s preference in other hospitals [3–5]. A Dacron patch was used in the EVCPP and SAVE procedures. The position of the sutured Dacron patch is described in detail in Table 1. Concomitant procedures comprising sub-valvular procedure, valve surgery, and ablation were performed, if required.

**Table 1:** Baseline characteristics at LAD implantation

No	Age (years)	Sex	Body mass index (kg/m^2^)	Body surface area (m^2^)	Etiology	Prior SVR, additional procedures	Portion of Dacron Patch	Time between SVR and cf-LVAD implantation (months)	INTERMACS level	Temporary MCS
1	59	M	23	1.82	DCM	SAVE, MVP, TAP	Anterior to septum	71	3	–
2	39	M	18	1.63	DCM	RPR + PM approximation, MVP, TAP	No	39	3	–
3	61	M	20	1.66	DCM	EVCPP, MVP	Apical	68	2	IABP
4	44	M	24	1.92	ICM	EVCPP, MVP, TAP	Apical	37	2	–
5	56	F	22	1.48	DCM	EVCPP, MVP, PVI	Apical	120	3	–
6	41	M	19	1.46	DCM	RPR + PM approximation, MVP, TAP	No	111	3	–

M, male, F, female, DCM, dilated cardiomyopathy, ICM, ischemic cardiomyopathy, SVR, surgical ventricular restoration, SAVE, septal anterior ventricular exclusion, MVP, mitral valve plasty, TAP, tricuspid annular plasty, PRP, posterior restoration procedure, PM, papillary muscle, EVCPP, endoventricular circular patch plasty, PVI, pulmonary vein isolation, cf-LVAD, continuous flow left ventricular assist device, INTERMACS, Interagency Registry for Mechanically Assisted Circulatory Support, MCS, mechanical care support, IABP, intra-aortic balloon pumping

### LVAD implantation surgery after left ventricular restoration

All LVAD implantation procedures were performed via median sternotomy or left thoracotomy (LT) on cardiopulmonary bypass (CPB) on a beating heart. CPB is established by aortic cannulation in the ascending aorta or the proximal arch and venous cannulation via the SVC and IVC. In the LT approach, skin-incision in the sixth intercostal space was performed after establishing CPB through femoral artery and vein cannulation. First, an LVAD inflow cannula was implanted. The optimal position of the LVAD inflow cannula was estimated by finger palpation or using epicardial echocardiography. Subsequently, coring of the left ventricular wall was performed by avoiding the sutured line created at the previous SVR procedure. An LVAD apical cuff was sutured onto the cored hole. An outflow graft was sutured end-to-side to the proximal ascending aorta in cases with a median sternotomy approach, or the descending aorta in the case of the LT approach. A pump pocket or space was created according to the size of each device and the anatomical position of the cored area.

### Statistical analysis

Results are expressed as mean ± standard deviation (SD) or as median + 25th–75th percentile interquartile range for continuous variables and frequency and percentage for categorical variables, as appropriate. The Friedman test was used to compare the three paired groups with nonparametric nature of data. Data for survival and freedom from cardiac events and all-cause death were derived using a Kaplan–Meier method; comparisons were made using a log-rank test. A two-sided *p* value of < 0.05 was considered statistically significant. All statistical analyses were performed using R (The R Project for Statistical Computing; The R Foundation).

## Results

### Patient characteristics

A total of six patients [5 male and 1 female, median age: 50 years (IQR: 42–58 years)] with a history of SVR surgery, who underwent cf-LVAD implantation in our institution due to recurrent severe heart failure were included in the study. The median interval between SVR and cf-LVAD implantation was 70 months (IQR: 46–101 months). Based on the clinical/medical history and muscle biopsy histology, the etiologies of cardiomyopathies consisted of idiopathic dilated cardiomyopathy (*n* = 5) and ischemic cardiomyopathy (*n* = 1). Previously performed SVR was classified into three types composing of the EVCPP procedure (*n* = 3), PRP procedures (*n* = 2), and SAVE procedure (*n* = 1). All SVR procedures were performed through a median sternotomy. At the time of the cf-LVAD implantation, the patients had a median INTERMACS level 3 (IQR: 2–3). Only one patient (INTERMACS level 2) required intra-aortic balloon pumping before cf-VAD implantation, while the other five patients required no temporary mechanical circulatory support. Additionally, all patients required high-dose inotropes, but no patient needed intubation. Baseline characteristics at cf-LVAD implantation are shown in Table 1.

### Left ventricular dimension and hemodynamic state before cf-LVAD implantation

The values of thoracic echocardiography and right heart catheterization before cf-LVAD implantation are summarized in Table 2. The median left ventricular ejection fraction was 16% (IQR; 12–23, range: 10–35). The median left ventricular diastolic diameter and systolic diameter were 73 mm (IQR; 68–82, range: 64–85) and 68 mm (IQR; 64–74, range: 53–80), respectively. The median left ventricular end-systolic volume index (LVESVI) was 154 ml/m^2^ (IQR; 136–170, range: 64–267), and only one patient (patient No. 4) had LVESVI under 70 ml/m^2^. The median mean pulmonary capillary wedge pressure (PCWP) was 24 mmHg (IQR; 21–27, range: 17–30), and five patients (except patient No. 5) had higher values than 20 mmHg. The median cardiac index (CI) was 1.7 L/min/m^2^ (IQR; 1.6–1.9, range: 1.5–2.2), and all patients had CI values lower than 2.2 L/min/m^2^. The median pulmonary vascular resistance (PVR) was 4.3 Wood units (IQR; 3.5–5.1, range: 2.4–6.9, this was not measured for patient No. 1), and three patients (No. 2, 3, and 4) had PVR ≥ 4.0 Wood units.

**Table 2:** Parameters of echocardiography and heart catheterization at cf-LVAD implantation

No	LVEF (%)	LVDd (mm)	LVDs (mm)	LVEDV (ml)	LVESV (ml)	LVESVI (ml/m^2^)	SVI (ml/m^2^)	mPCWP (mmHg)	CI (L/min/m^2^)	PVR (wood)
1	24	85	75	394	301	165	51	20	1.53	–
2	20	72	65	273	219	134	33	22	2.2	4.26
3	12	84	80	503	442	267	37	30	1.54	6.91
4	35	64	53	160	123	64	19	27	1.7	5.06
5	4	62	61	260	225	152	24	17	1.7	3.46
6	10	67	64	231	209	144	15	26	2	2.41

LVEF, left ventricle ejection fraction, LVDd, left ventricular end-diastolic diameter, LVDs, left ventricular end-systolic diameter, LVEDV, left ventricular end-diastolic diameter, LVESV, left ventricular end-systolic diameter, LVESVI, left ventricular end-systolic volume index, SVI, systolic volume index, mPCWP, mean pulmonary capillary wedge pressure, CI, cardiac index, PVR, pulmonary vascular resistance

### Perioperative results and follow-up

All patients underwent cf-LVAD implantation as BTT strategy. First, one patient (No. 1) underwent cf-LVAD implantation with left thoracotomy due to strong adhesion of the left ventricular anterior lesion caused after the SAVE operation. Median sternotomy was chosen for the other five patients because of severe focal adhesion of the left ventricular apical or posterior region. Implanted devices [Jarvik 2000 (*n* = 2), HM2 (*n* = 1), EVAHEART II (*n* = 1), DuraHeart (*n* = 1), and HVAD (*n* = 1), respectively] and the concomitant valve surgery are described in Table 3. In one patient (No. 1), after implanting Jarvik 2000 through the LT approach, the outflow graft was sutured on the descending aorta.

**Table 3:** Intraoperative findings

No.	Heart transplant strategy	Approach	Outflow graft anastomosis	LVAD type	Additional procedures	Cardiac arrest or beating	Removal of Dacron patch
1	BTT	left thoracotomy	Descending aorta	Jarvik 2000		Beating	No-removal
2	BTT	Median sternotomy	Ascending aorta	EVAHEART	–	Beating	–
3	BTT	Median sternotomy	Ascending aorta	HeartMate II	Tricuspid annular plasty	Beating	No-removal
4	BTT	Median sternotomy	Ascending aorta	DuraHeart	Tricuspid annular plasty	Beating	No-removal
5	BTT	Median sternotomy	Ascending aorta	HVAD	–	Beating	No-removal
6	BTT	Median sternotomy	Ascending aorta	Jarvik 2000	–	Beating	–

BTT, bridge to transplant; LVAD, left ventricular assist device

Intraoperative echocardiography-guided epicardial images along with finger palpation were used to determine the optimal position of the inflow cannulation site. It was possible to place an inflow cannula in the left ventricular anterior portion (at the level of the transition zone between the sutured line and the myocardium), at least 2 cm apart from the sutured line created in the EVCPP procedures and the optimal left ventricle-apical portion in the PRP and SAVE procedures. Removal of the implanted Dacron Patch or additional left ventricle plasty was not performed. No patient experienced any perioperative complications such as re-thoracotomy or cardiac tamponade. Various type of VAD associated complications were observed in all patients during a follow-up, but, due to no major sequelae, any patient was not excluded from a heart transplantation candidate (Table 4). Three patients (No. 1, 2, and 6) finally underwent orthotropic heart transplantation after a waiting time of 39, 56, and 61 months, respectively. The remaining three patients (No. 3, 4, and 5) are still on the device in good clinical condition after 40, 75, and 11 months, respectively (Table 3). At present, all patients are alive at median 67 months (IQR: 41–93 months, range 4–155 months) after cf-LVAD implantation.

**Table 4:** Outcomes after LVAD therapy

No	Time on device (months)	VAD-associated complications	Outcomes	Time after heart transplant (months)	Survival
1	39	Drive-line infection	Heart transplant	115	Alive
2	56	Non-disable cerebrovascular accident	Heart transplant	44	Alive
3	40	Disable cerebrovascular accident	On waiting list	–	Alive
4	75	Non-disable cerebrovascular accident	On waiting list	–	Alive
		Drive-line infection			
5	11	Increased aortic insufficiency	On waiting list	–	Alive
6	61	Drive-line infection	Heart transplant	5	Alive

VAD, ventricular assist device

### Hemodynamic change

The hemodynamic value was determined at pre-LVAD implantation, 1 month post-VAD-implantation, and during the late follow-up after LVAD implantation (median 39 months, IQR; 35–55, range 10–71 months; Table 5). The mean value of PCWP after LVAD implantation significantly decreased as compared to that before LVAD implantation (p < 0.05), and the cardiac index after LVAD implantation increased (p < 0.05). PVR after LVAD support was significantly improved to a normal range.

**Table 5:** Hemodynamic change at pre-VAD implantation, 1-month LVAD implantation, and at late follow-up

	Pre VAD	1 month VAD	Late-follow-up VAD*	*p* value
Mean pulmonary artery pressure (mmHg)	37 (IQR 33–44, range 26–49)	18 (IQR 18–23, range 14–27)	23 (IQR 21–27, range 18–30)	0.038
Mean pulmonary capillary wedge pressure (mmHg)	24 (IQR 21–27, range 17–30)	11 (IQR 9–12, range 8–17)	11 (IQR 9–15, range 8–17)	0.034
Cardiac index (L/min/m2)	1.7 (IQR 1.58–1.93, range 1.53–2.2)	2.56 (IQR 2.32–2.76, range 2.12–2.94)	2.76 (IQR 2.36–3.03, range 1.82–3.11)	0.016
Mean right atrium pressure (mmHg)	8.5 (IQR 7.25–12.8, range 2–22)	9 (IQR 7–11, range 2–13)	8 (IQR 8–9, range 7–14)	0.49
Pulmonary vascular resistance (Wood)	4.26 (IQR 3.46–5.06, range 2.41–6.91)	1.83 (IQR 1.81–2.7, range 0.48–2.7)	1.83 (IQR 1.71–3.53, range 1.6–4.04)	0.038

^*^Late follow-up duration was median 39 months (IQR; 35–55, range 10–71 months)

## Discussion

We successfully implanted five types of cf-LVAD in patients who had undergone SVR, including EVCCP, SAVE, and PRP with pupillary muscle approximation. To our knowledge, this is the first study to report the results after LVAD implantation following various types of SVR.

Cf-LVAD implantation improves the survival and quality of life of patients with heart failure. However, there are limited reports of VAD therapy in patients with prior SVR surgery. Therefore, the aim of this study was to assess feasibility of implantation surgery and the incidence of complications, outcomes, and safety of cf-LVAD therapy in this cohort. Several factors can make cf-LVAD implantation after SVR extremely challenging, such as the altered apical anatomy and the presence of an endoventricular patch.

Previously, Palmen et al. published a case series describing their experience in seven patients with a history of SVR (EVCPP procedure) who had VAD implants (HVAD, Medtronic) [14]. After a follow-up of 20 ± 16 months, they reported that all seven patients were alive without any VAD complication. Interestingly, the Dacron patch implanted in the EVCPP procedure was completely removed with cardiac arrest in all patients, because the remaining left ventricular cavity was estimated to be too small to accommodate the inflow cannula of the LVAD without interference with LVAD inflow and suction of the mitral valve apparatus. The limitations in their study were as follows: (1) A prior SVR was limited to the EVCPP procedure, (2) only HVAD was implanted, and (3) the follow-up period after LVAD implantation was short. In our cohort, the patients underwent all types of SVR, including SAVE with an anteroseptal Dacron patch, EVCPP with a patch in apical portion, and PRP with pupillary muscle approximation. Additionally, implanted LVAD types composed of five device types (Table 3). The follow-up time (median 39 months (IQR; 35–55, range 10–71 months)) in our cohort was longer; wherein all patients survived and three (50%) patients could finally receive heart transplantation. As a result, our patients had effective left ventricle unloading and the long durability in failed SVR (Table 5).

While removal of the endoventricular Dacron patch may be effective in improving the position of the inflow cannula, it is not always necessary for LVAD implantation [15]. We did not need to remove the Dacron patch in patients after SAVE and EVCPP procedures. Most patients with recurrent symptomatic heart failure after failed SVR showed dilated left ventricular dimension again (Table 2). Due to the recurrent dilated left ventricular dimension, the optimal inflow cannula position could be easily explored by direct epicardial and transoesophageal echocardiography, and device apical cuff anastomosis could be decided. Moreover, epicardial echocardiography could increase the success rate of this procedure [13]. However, in the above-described fashion, we could implant two types of LVADs (EVAHEART and Jarvik 2000) in left ventricle positions to allow acceptable circulatory flow.

Leaving the Dacron patch in place seems to have some advantages. First, the LVAD implantation is performed with a beating heart in the routine fashion. Therefore, the surgical procedure is not complicated and is quicker. Second, myocardium damage can be reduced. This can reduce the frequency of postoperative atrial or ventricular arrhythmia, and may decrease the risk of right heart failure. Third, the left ventricular hole after the removal of Dacron patch is too large and the tissue around the hole is fragile. Therefore, there may be difficulty in controlling the bleeding around the inflow cannula. For these reasons, we routinely performed both intraoperative transoesophageal and epicardial echocardiography in all cases in order to determine the optimal position of the inflow cannula. As a result, we could perform cf-LVAD implantation in a safe fashion (Supplement 1).

Careful attention should be paid while deciding the appropriate surgical strategy. If preoperative TTE and chest CT with contrast show signs of wedge thrombus around the apical area or Dacron patch, removing the Dacron patch and Fontan stitch may be a choice of optimal surgical strategy for an inspection of the left ventricular cavity.

### Limitations

There are several limitations to our study. This was a retrospective, single-center study with a limited number of patients. Even though this study does not provide generalized statements, we were still able to report good outcomes for this cohort.

## Supplementary Information

Below is the link to the electronic supplementary material.

Supplementary file1 (DOCX 16 KB)

## Data Availability

The data that support the findings of this study are available from the corresponding author, Takayuki Gyoten, upon reasonable request.
