# Supplementary material for: Outcomes of continuous flow left ventricular assist device after surgical left ventricular restoration
Source: Gen Thorac Cardiovasc Surg. 2023 Feb 21;71(8):480–6. doi: 10.1007/s11748-023-01917-8 (PMC10344836; doi:10.1007/s11748-023-01917-8)
Supplement: Supplementary file 1 — Supplementary file1 (DOCX 16 KB) [file 11748_2023_1917_MOESM1_ESM.docx]

|  | SVR group (n=6) | Non-SVR group (n=184) | P-value |
| --- | --- | --- | --- |
| Operative time, minute | 396 (379-408) | 428 (338-472) | 0.52 |
| Cardiopulmonary bypass time, minute | 122 (119-123) | 141 (124-165) | 0.16 |
| Red cell concentrate, unit | 6 (5-6) | 6 (4-12) | 0.53 |
| Fresh frozen plasm, unit | 6 (5-7) | 6 (4-8) | 0.68 |
| Platelet, unit | 20 (10-30) | 20 (0-20) | 0.49 |
| Intensive care unit stay, day | 3 (2-5) | 5 (4-7) | 0.14 |
| Survival at 30 days | 100% | 99% | n.p. |

　　　　 Supplement 1. Perioperative outcomes between the SVR group and the non-SVR cohort.
